# Rugby Fans in Training New Zealand (RUFIT-NZ): a pilot randomized controlled trial of a healthy lifestyle program for overweight men delivered through professional rugby clubs in New Zealand

**DOI:** 10.1186/s12889-019-6472-3

**Published:** 2019-02-08

**Authors:** Ralph Maddison, Elaine Anne Hargreaves, Sally Wyke, Cindy M. Gray, Kate Hunt, Justin Ihirangi Heke, Stephen Kara, Cliona Ni Mhurchu, Andrew Jull, Yannan Jiang, Gerhard Sundborn, Samantha Marsh

**Affiliations:** 1 Institute for Physical Activity and Nutrition (IPAN), School of Exercise and Nutrition Sciences, Deakin University, 221 Burwood Highway, Burwood, Melbourne, Vic 3125 Australia; 2 School of Physical Education, Sport & Exercise Sciences, University of Otago, Dunedin, New Zealand; 3 Institute of Health and Wellbeing, College of Social Sciences, University of Glasgow, Glasgow, Scotland, UK; 4 Institute for Social Marketing, Faculty of Health and Sports Sciences, University of Stirling, Stirling, UK; 5 Integrated Hauora Initiatives Ltd, Wellsford, New Zealand; 6 Axis Sports Medicine Clinic, Auckland, New Zealand; 7 National Institute for Health Innovation, University of Auckland, Auckland, New Zealand; 8 Epidemiology and Biostatistics, School of Population Health, University of Auckland, Auckland, New Zealand

**Keywords:** Physical activity, Obesity, Weight management, Diet, Men’s health, Lifestyle intervention, Professional sports clubs

## Abstract

**Background:**

Healthy lifestyle programs that are designed specifically to appeal to and support men to improve lifestyle behaviors and lose weight are needed. The Rugby Fans in Training-New Zealand (RUFIT-NZ) program is delivered by professional rugby clubs and inspired by the successful Football Fans In Training program (FFIT), a gender sensitized weight loss program for obese middle-aged men delivered by professional football clubs in Scotland. RUFIT-NZ required development and evaluation for feasibility.

**Methods:**

To develop the intervention we reviewed content from the FFIT program and evidence-based physical activity, dietary and weight management guidelines, and undertook a series of focus groups and key informant interviews. We then evaluated the feasibility of the intervention in a two-arm, parallel, pilot randomized controlled trial in New Zealand. Ninety-six participants were randomized to either the 12-week RUFIT-NZ intervention (*N* = 49) or a control group (*N* = 47). The intervention was delivered through professional rugby clubs and involved physical activity training and classroom sessions on healthy lifestyle behaviors. Pilot trial outcomes included body weight, heart rate, blood pressure, cardiorespiratory fitness, and lifestyle behaviors. Feasibility was assessed by recruitment and retention rates, and acceptability of the intervention.

**Results:**

At 12 weeks the mean difference in body weight was 2.5 kg (95% CI -0.4 to 5.4), which favored the intervention. Statistically significant differences in favor of the intervention group were also observed for waist circumference, resting heart rate, diastolic blood pressure, cardiorespiratory fitness, and the proportion of participants that were adherent to 3 or more healthy lifestyle behaviors. The intervention was considered feasible to test in a full trial given the good recruitment and retention rates, and positive feedback from participants.

**Conclusions:**

A pilot study of a healthy lifestyle intervention delivered via professional rugby clubs in New Zealand demonstrated positive effects on weight and physiological outcomes, as well as adherence to lifestyle behaviors. Feasibility issues in terms of recruitment, retention, and participant acceptability were assessed and findings will be used to inform the design of a definitive trial.

**Trial registration:**

The trial was prospectively registered with the Australian New Zealand Clinical Trials Registry ACTRN12616000137493, 05/12/2016.

## Background

In New Zealand (NZ), 30% of men are obese and a further 39% are overweight, with significantly higher rates among Māori (Indigenous New Zealanders) and Pacific men (adjusted rate ratios 1.7 for Māori vs non- Māori, and 2.4 for Pacific vs non-Pacific) [1]. Men also have a lower life expectancy than women, despite having comparable self-reported ratings of good to excellent health [1]. Reasons for this are multifactorial, but include unhealthy diet, physical inactivity and the high prevalence of obesity, as well as past rates of tobacco smoking [2].

Despite common stereotypes to the contrary, research has shown that men are concerned about their weight [3] and health [4, 5]; however engaging men in existing commercial or health service-based weight management programs is difficult [6] (e.g., between 80 and 90% of commercial program attendees are female) [7–9]. Reasons for lack of participation include men’s perceptions that dieting and weight management programs are ‘for women’ [3, 10], as well as concerns about feeling out of place in female-dominated groups [11]. Other reasons for low attendance at existing programs include men not being aware they are overweight, not knowing about weight loss programs or how to lose weight, not wanting to ask for help, and perceptions of embodied masculinities that make them value bigger, muscular bodies [12]. Given men’s concerns regarding their weight [3] and health [4, 5], they may welcome a tailored healthy lifestyle program [6]. A healthy lifestyle program that is appealing to men while also supporting them in weight loss and long-term lifestyle changes is urgently needed to improve the health of NZ men.

A systematic review and meta-analysis of male-only weight loss programs reported a significant difference in weight change favouring weight loss interventions over no-intervention controls at the last reported assessment (weighted mean difference of − 5.66 kg, 95% CI − 6.35, − 4.97). Characteristics common to effectiveness were being younger (mean age ≤ 42.8 years), increased frequency of contact (> 2.7 contacts/month), group face-to-face contact and inclusion of a prescribed energy restriction [13]. However, the studies included in this meta-analysis were generally low quality, with small sample sizes, lack of blinding, had issues with allocation concealment, and a lack of intention-to-treat (ITT) analysis. A 2014 systematic review, which assessed the quantitative (33 randomized controlled trials [RCTs with 12 linked reports, and 24 non-randomized reports]), qualitative (22 qualitative studies), and economic evidence base (5 economic evaluations with 2 linked reports) for the management of obesity in men described similar effects, with interventions that combined a low-fat diet with physical activity and behavior change training demonstrating the greatest reduction in weight after 4 years (5.2 kg; SE 0.2) [14]. This review also highlighted two important issues regarding the effectiveness of these programs: (1) men were more likely than women to benefit if there was a physical activity component included; and (2) the addition of diets focused on reduction of energy intake tended to result in more favorable weight loss outcomes than programs that focused on physical activity alone. Consistent with weight management guidelines, programs that included physical activity, a diet component, and behavior change strategies demonstrated the most effective results [14]. In terms of engaging men, the review showed that although fewer men joined weight-loss programmes, once recruited they were less likely to drop out than women (difference 11, 95%CI 8 to 14%). Factors related to engagement included having the perception of having a health problem, the impact of weight loss on health problems and desire to improve personal appearance. Generally, men preferred more factual information on how to lose weight and more emphasis on physical activity programs [14]. These latter findings are consistent with Morgan et al., [6] who reported that men were attracted to a program that was tailored for them and did not require extensive time commitments. They also valued education about energy balance and the use of humour to deliver simple messages [14].

In the United Kingdom, English Premier League football clubs have successfully engaged men in various health initiatives since 2009 [15, 16]. Although largely unstructured and lacking rigorous systematic evaluation, process evaluations of these programs have shown positive results. In Scotland, the Football Fans In Training (FFIT) program, a weight management and healthy lifestyle program targeted at middle-aged men (aged 35–65 years) who were classed as overweight or obese [BMI 28 kg/m^2^ or above], was developed for delivery by community coaching staff at professional football clubs [17]. FFIT an evidence-based, gender-sensitized in context, content and style of delivery, included behavior change techniques known to be effective in promoting weight loss and physical activity [18, 19], and components designed to improve healthy eating, physical activity, and alcohol consumption [20]. A pragmatic RCT of FFIT (*n* = 747) showed a mean difference in weight loss of 4.94 kg (95% CI 3.95–5.94) at 12 months, favoring the intervention group (after adjustment for delivery club and baseline weight) [21]. Similar effects were found for the adjusted percentage weight loss at 12 months (4.36, 95% CI 3.64–5.08), again in favor of the intervention group. A process evaluation found that the football club proved a powerful ‘draw’ to the program, both symbolically and physically and that the program attracted men at risk of ill health, who had wanted to make changes but had been reluctant to attend existing weight management programs [11, 22]. It also suggested that interaction with other men during the program allowed men not only to think differently, but also to support each other in making changes to their health practices. That is, participation in the 12-week program enabled men to enact a changed lifestyle and allowed them to re-negotiate aspects of their gendered performances and behavior in relation to health [11, 22–24]. These findings highlight the growing interest in harnessing the attraction of professional sports clubs to encourage men to participate in a range of health promotion initiatives [11, 17, 21, 25].

We considered that a program inspired by FFIT, but conducted through professional rugby clubs, could be effective and feasible for targeting overweight and obese males in NZ. The program was named Rugby Fans in Training NZ (RUFIT-NZ). Rugby (Union and League) is an integral part of NZ culture, the most popular spectator team sport, with high participation rates, particularly among Māori and Pacific peoples. A gender-sensitive lifestyle intervention program that harnesses the popularity of rugby and the culture of ‘male masculinity’ surrounding it, [26] may therefore help target this underserved group by addressing perceived barriers to participation (e.g. preoccupations with weight loss and dieting being women’s issues) [12]. Further, this approach capitalizes on the traditional male sporting environment, the powerful social and psychological connection to the sports team (e.g., loyalty, identity) that being a fan creates, and the opportunity for men-only support [27].

Whilst FFIT was effective both for weight loss and engaging men aged 35–65 years through football in Scotland, in developing the NZ program we were cognizant that changes to the program may be needed to align with the rugby environment and cultural needs of men in NZ. We reasoned that some changes may need to be made before the same benefits could be gained through participation in the RUFIT-NZ program. FFIT’s generalizability to different ethnic groups and other sports has yet to be determined; however it is currently being explored elsewhere, e.g. in a pilot study with Canadian Ice Hockey [28]. Therefore, prior to conducting a larger RCT to determine the effectiveness of the RUFIT-NZ program in supporting weight loss and behavior change in men, the feasibility and acceptability of such a program first needs to be assessed. Thabane et al. [29] recommend pilot studies as a pre-requisite for the assessment of feasibility prior to full-scale studies.

The aims of this research were to: 1) develop the RUFIT-NZ program to be delivered via professional rugby clubs; 2) explore the potential for RUFIT-NZ to help overweight or obese men lose weight and make healthy lifestyle changes by 12 weeks; and 3) evaluate the feasibility of conducting a definitive trial of the RUFIT-NZ program, as assessed by recruitment rates, participant retention, and participant feedback.

## Methods

### Development of the intervention

A formative process was used to develop the RUFIT-NZ intervention to ensure it would resonate with NZ men. First, we reviewed NZ-based guidelines for weight management, physical activity and diet in adults to ensure alignment. NZ weight management guidelines [30] promote food, activity and behavioral support (FAB) principles. Second, we reviewed content from the original FFIT program [21, 22]. Third, focus groups and interviews with key stakeholders were conducted to guide the intervention content and features to promote sustainability of the program. The objectives of the focus groups were to gain in-depth information about what potential participants wanted from the RUFIT-NZ program, whether, and if so how, wives and partners of study participants should or could be included in the program, and the optimal ways to deliver the program. Overweight men interested in sharing their views in a focus group setting responded to recruitment advertise-ments. Five focus groups (*n* = 47) were conducted with men (2 in Dunedin and 3 in Auckland) recruited via existing networks. Female participants (*N* = 17, 2 focus groups) were recruited either through the partners who participated in the male focus groups or through existing networks. A semi-structured discussion guide was created to direct the focus group discussion. This comprised key questions to initiate discussion on the focus group objectives but also to allow further unscripted questioning based on how the discussions unfolded. The focus groups were facilitated by trained researchers. Thematic analysis was used to identity key themes emerging from the data [31]. Seven focus groups resulted in data saturation so no further groups were recruited. Overall, focus groups identified the need for RUFIT-NZ, like FFIT, to have a more holistic approach to health, rather than focus on weight alone. Participants identified with the idea of delivering a healthy lifestyle program via professional clubs but stated it should be free of charge and available at times that meet the needs of potential participants (later on weekdays or on weekends). Involving family members and including culturally appropriate content was considered important by Pacific men and women, but not others.

Interviews (*N* = 5) with key stakeholders were conducted to ensure engagement with existing healthcare and health promotion agencies in NZ. Five key stakeholder groups were identified as being able to provide relevant and strategic opinion, these were Green Prescription, Public Healthcare Organizations, and Māori and Pacific healthcare providers. The objectives of the interviews were to gain information on the proposed program, possible approaches to recruit or engage participants, and to identify ways of referring men identified at risk for chronic disease. Each interview was directed by a semi-structured interview guide which ensured the objectives were addressed, while also allowing the stakeholder to discuss other topics they believed important. Participant safety was a common theme raised by stakeholders, highlighting the need to screen overweight or obese participants for other co-morbidities (such as high blood pressure, diabetes etc.) and to consider a mechanism for referring at-risk participants to their primary care physician. A second theme was the need to ensure RUFIT-NZ included content delivered by existing healthcare providers, such as Pacific Island Heartbeat (a Heart Foundation endorsed program to promote healthy eating for Pacific families). In line with this, it was felt that written resources should reflect cultural values by including common Māori or Pacific terminology. A third theme related to the sustainability of RUFIT-NZ beyond the conduct of an RCT. Stakeholders discussed the need to find a suitable funding or business model to ensure the sustained viability of the program.

Collating all information, the research team developed the RUFIT-NZ intervention, which while inspired by FFIT, included some differences (in eligibility, the intervention itself, and specific behavior change techniques [BCTs) recognized as key facilitators of physical activity and dietary behavior change intervention effectiveness [18], which are presented in Tables 1 and 2.

**Table 1:** The RUFIT-NZ program (Auckland and Dunedin) compared with the FFIT program

	RUFIT-NZ		FFIT
	Auckland	Dunedin	Scotland
PARTICIPANTS			
*Overall goal of program*	Improve health through lifestyle changes	Improve health through lifestyle changes	Getting fitter, losing weight and feeling better
*Inclusion/exclusion criteria*	Age = 25–65 years BMI = ≥25 kg/m^2^ Completed PAR-Q^a^ Not meeting PA guidelines Provided informed consent	Age = 25–65 years BMI = ≥25 kg/m^2^ Completed PAR-Q^a^ Not meeting PA guidelines Provided informed consent	Age = 35–65 years BMI = ≥28 kg/m^2^ Completed PAR-Q^b^ Provided informed consent Men with systolic BP ≥160 mmHg or diastolic BP ≥100 mmHg excluded from intense PA until evidence provided of reduction in BP.
*Maximum no. of men in group*	*N* = 20	*N* = 30 (maximum of 12–13 per group)	*N* = 30 (with a maximum coach: participant ratio of 1:15)
INTERVENTION			
Intensity			
*No. of sessions*	24 sessions (1 x PA session and 1 x PA + classroom session per week^c^)	12 sessions (1 x PA session + classroom session per week^c^)	12 sessions (1 x PA + classroom session per week)
*Duration of sessions*	90 min for both physical and classroom sessions (Total 30 h over 12 weeks)	120–150 min (Total 24-30 h over 12 weeks)	90 min (Total 18 h over 12 weeks)
Content			
*Classroom*			
☐ Introduction from trainer/coach	✓	✓	✓
☐ Getting to know one another	✓	✓	✓
☐ Health benefits of weight loss	✓	✓	✓
☐ Nutrition			
○ *Inclusion of partners to nutrition session*	✓	✗	✗
○ *Energy balance*	✓	✓	✓
○ Food choices	✓	✓	✓
○ Food groups	✓	✓	✓
○ Healthy eating plans	✓	✓	✓
○ Food labels	✓	✓	✓
○ Eating out	✓	✓	✓
○ Food diaries	✗	✗	✓
○ Mindful eating	✓	✓	✗
○ Sugary drinks	✓	✓	✓
☐ Alcohol	✓	✓	✓
☐ BCTs (see Table 2)	✓	✓	✓
☐ Health benefits of PA	✓	✓	✓
☐ Sleep	✓	✓	✗
☐ Sedentary behavior/screen use	✓	✓	✗
☐ Myth busting	✓	✓	✓
☐ Long-term maintenance	✓	✓	✓
*Physical activity*			
☐ Component 1: Pedometers	Incremental pedometer-based daily walking program	Incremental pedometer-based daily walking program	Incremental pedometer-based daily walking program
☐ Component 2: PA sessions	Gym-based and field-side sessions. First 4 weeks predominantly aerobic, off-feet training and body weight exercises, second 4 weeks introduce external loads and increase running volume, last 4 weeks introduce strength, aerobic, and anaerobic conditioning.	Gym-based and field-side sessions. First 4 weeks predominantly aerobic, off-feet training and body weight exercises, second 4 weeks introduce external loads and increase running volume, last 4 weeks introduce strength, aerobic, and anaerobic conditioning.	Pitch-side/in-stadia PA sessions, with men trained to work at their own optimal level of intensity as assessed by the Rate of Perceived Exertion Scale.
Delivery			
*Staff*	Club trainer, club doctor, club nutritionist, and community nutrition group (no formal training given)	Community coach, club doctor, club nutritionist, and community dietician (no formal training given)	Trained community coaching staff
*Delivery of classroom sessions*			
☐ Power point presentations	✓	✓	✗
☐ Supportive group environment	✓	✓	✓
☐ Sharing of experiences	✓	✓	✓
☐ Interactive problem solving	✓	✓	✓
☐ Repeated practice of BCTs	✓	✓	✓
☐ Coaches available at end of each session	✓	✓	✓
*Balance of PA and classroom sessions*	PA and classroom sessions balanced throughout the program	PA and classroom sessions balanced throughout the program	Balance of PA and classroom sessions changed throughout the program (later weeks focused more on PA with shorter classroom sessions)
MAINTENANCE	None as only a pilot trial of 12 weeks duration	None as only a pilot trial of 12 weeks duration	‘Light touch’ weight maintenance phase = 6 post-program email prompts over 9 months and a group reunion at 6 months

*PAR-Q* physical activity readiness questionnaire, *PA* physical activity, *BCTs* behavioral change techniques

^a^Physician consent to participate required for all participants who responded ‘Yes’ to any PAR-Q items

^b^Those with high blood pressure or other contraindications to vigorous physical activity were able to take part in classroom session of FFIT and in the graduated pedometer-based walking program, but were not able to participate in more vigorous group physical activity sessions until they could provide evidence that their contraindication was resolved, but physician consent to participate was not required for participants endorsing any PAR-Q items

^c^Classroom sessions were not always delivered every week (e.g. some weeks just included physical activity sessions)

**Table 2:** List of Behavior Change Techniques utilized in RUFIT-NZ and FFIT by grouping (BCT Taxonomy v1) [44]

BCT Grouping	BCT	BCTs utilised by RUFIT-NZ^a^ and FFIT	
		RUFIT-NZ	FFIT
1. Goals and planning	1. Goal setting (behavior)	✓	✓
	2. Goat setting (outcome)	✓	✓
	3. Behavioral contract	✓	✓
	4. Commitment	✓	✓
	5. Action planning (includes Implementation intentions)	✓	✓
	6. Review behavior goals	✓	✓
	7. Review outcome goal(s)	✓	✓
	8. Discrepancy between current behavior and goal	✓	✓
	9. Problem solving (includes Relapse prevention)	✓	✓
2. Feedback and monitoring	2.1. Feedback on behavior	✓	✓
	2.2 Monitoring of behavior by others (coaches) without feedback	✓	✗
	2.3. Feedback on outcome(s) of behavior	✗	✓
	2.4. Self-monitoring of behavior	✓	✓
	2.5. Self-monitoring of outcome(s) of behavior	✓	✓
3. Social support	3.1. Social support (unspecified)	✓	✓
	3.2. Social support (practical)	✓	✗
	3.3. Social support (emotional)	✓	✓
4. Shaping knowledge	4.1. Information about antecedents	✓	✓
	4.2. Re-attribution	✓	✓
	4.3. Instruction on how to perform a behavior	✓	✓
5. Natural consequences	5.1. Information about health consequences	✓	✓
	5.2. Salience of consequences	✓	✓
	5.3. Monitoring of emotional consequences	✗	✓
	5.4. Information about emotional consequences	✓	✓
6. Comparison of behavior	6.1. Social comparison	✓	✓
	6.2. Demonstration of the behavior	✓	✓
7. Comparison of outcomes	7.1. Persuasive source	✓	✓
8. Repetition and substitution	8.1. Behavioral practice/rehearsal	✓	✓
	8.2. Habit formation	✓	✓
	8.3. Behavior substitution	✓	✓
	8.4. Generalisation of a target behavior	✓	✓
	8.5. Graded tasks	✓	✓
	8.6. Habit reversal	✓	✗
9. Regulation	9.1. Reduce negative emotions	✓	✓
10. Antecedents	10.1. Avoidance/reducing exposure to cues for the behavior	✓	✓
	10.2. Adding objects to the environment	✓	✓
	10.3. Restructuring the social environment	✓	✓
11. Identity	11.1. Identification of self as a role model	✓	✗
	11.2. Framing/reframing	✓	✓
12. Self-belief	12.1. Verbal persuasion about capability	✓	✓
	12.2. Focus on past successes	✓	✓
13. Covert learning	13.1 Vicarious consequences	✗	✓

^a^RUFIT-NZ = both the Auckland and Otago programs

### Evaluation of the intervention

#### Methods and design

A two-arm parallel design pilot RCT was conducted, which was registered on the Australian and NZ clinical trials registry (ACTRN12616000137493). Data collection was conducted at baseline and at the end of the 12-week program. The trial was based in two professional rugby clubs who participate in the Super 18 rugby competition: (1) The Blues rugby club in Auckland (North Island), and (2) The Pulse Energy Highlanders in Dunedin (South Island), NZ. In Auckland, baseline and follow-up measures were assessed at the Blues rugby club, while in Dunedin they were assessed in a laboratory within the School of Physical Education, Sport & Exercise Sciences, University of Otago, as club facilities could not be accessed for this purpose.

The intervention is described according to the Consolidated Standards of Reporting Trials (CONSORT) 2010 statement: extension to randomized pilot and feasibility trials [32, 33]. The study was conducted in accordance with the Declaration of Helsinki and received approval from the University of Auckland Human Participants Ethics Committee (reference number 015069). The informed consent was obtained from all participants before they commenced the study.

#### Study population

Eligible participants were male adults aged 25–65 years who were overweight (defined as a BMI of ≥25 kg/m^2^), who self-reported not meeting the NZ physical activity guidelines (at least 2.5 h of moderate or 1.25 h of vigorous physical activity in a week [34]), able to safely undertake physical activity, able to understand and read English, and able to provide written informed consent to participate in the study. All participants were pre-screened using the Physical Activity Readiness Questionnaire (PAR-Q) [25], with physician consent to participate required for all participants who were identified as not safe to exercise. This step was highlighted in key informant interviews and was necessary to ensure that medical professionals deemed that men with pre-existing conditions could still participate in the program.

#### Recruitment

Recruitment for the trial commenced in February 2016. Participants were recruited via the participating clubs’ mailing lists, supporter registers, and Facebook pages. The study was also advertised using a Facebook-promoted post through the University of Auckland, and through a newspaper advertisement and front page article in Dunedin. Information sessions were also scheduled, where men could come along to a presentation about the program and ask any questions they may have. Men agreeing to participate were screened for eligibility by phone or email, given a study pack with a Participant Information Sheet and Consent Form, and then scheduled to attend a baseline assessment.

#### Randomisation

Participants were randomized to either the intervention or control group in a 1:1 ratio; the randomization sequence was generated by computer program using variable block sizes of 2 or 4, and overseen by the study statistician (YJ), stratified by study center. The group allocation was concealed until the point of randomization, using opaque sealed sequentially numbered envelopes prepared by a researcher not involved in the study. Randomization was performed prior to the baseline assessment. Due to the nature of the study, participants were aware of their treatment allocation. Outcome assessors were aware of treatment allocation but had no role in the intervention delivery.

#### Intervention

While inspired by FFIT [21, 22], the RUFIT-NZ program was modified in a number of ways (see Tables 1 & 2 for differences between the programs). In addition, as this study sought to address feasibility issues such as delivery of intervention content and frequency of available sessions, the research team chose to include core features common to both Auckland and Dunedin programs, but with some variation, which is described below. These variations were driven primarily for pragmatic reasons (time and available resources at each club) and allowed exploration of whether differences in delivery had an effect on key feasibility issues, including ease of recruitment and retention of participants.

The 12-week RUFIT-NZ intervention consisted of twice-weekly 90-min sessions in Auckland (one during the weekend and one during the working week) and a once-weekly 120–150 min session in Dunedin (held early evening during the working week), which were run at the respective rugby clubs by a RUFIT-NZ coach. In Auckland, the weekend session consisted of a 30-min classroom session followed by a 60-min physical activity session, while the session during the working week consisted of a 90-min physical activity session only. In Dunedin, the session comprised 60 min of physical activity and 60–90 min for the classroom session. The number of classroom sessions delivered was the same for the two clubs. The content of the classroom sessions was standardized, so that the men participating at both clubs received the same educational material. Following on findings from the focus groups, the program aimed to focus less on weight, but have a more holistic approach targeting a range of health-related behaviors (nutrition, physical activity, sleep, and sedentary behavior), thus classroom sessions covered information on SMART goal setting and other behavior change strategies.

The classroom sessions were run in an informal setting, where the participants sat together and had the opportunity to discuss the content of the session as a group. At the end of each classroom session, participants set a goal that was relevant to the content that was delivered during the session. They were encouraged to follow these goals outside of the structured program. In Auckland, three nutrition sessions were delivered by Pacific Heartbeat, a trained community nutrition education group aligned with the New Zealand Heart Foundation, while in Dunedin nutrition sessions were run by a community-registered dietician (holding a Masters degree in Dietetics). To enhance the connection to the rugby environment, in both settings, another nutrition session was run by the Rugby Club’s dietician and the session on alcohol consumption was delivered by the Club’s doctor. The RUFIT-NZ coach delivered the remaining education sessions. In Auckland, the RUFIT-NZ coach was a trainer for the rugby franchise and in Dunedin, the coach was a former player, now a qualified personal trainer. RUFIT-NZ coaches were qualified strength and conditioning trainers with greater than 5 years’ experience of delivering exercise training programs to men of varying fitness abilities. They were provided in-house training by the investigators (RM, EH, SM) on the process of delivering the RUFIT-NZ material prior to the start of the program.

Participants were supported to increase their physical activity in two ways: 1) through the coach-led physical activity session; and 2) all participants received a pedometer and an individualized weekly step goal program [35–37] to follow outside of the structured program. The physical activity program (designed by the club trainer) progressively increased in difficulty over 12 weeks. The first 4 weeks predominantly consisted of aerobic off-feet conditioning using equipment commonly available in rugby clubs in NZ (e.g. rower and bike) and body weight exercises (squats, push-ups, body weight rows, lunges and core work). The second 4 weeks consisted of the same exercises as above but also introduced external loads (e.g. kettle bells, dumbbells, barbells etc.), and running volume increased according to each participants fitness level. The last 4 weeks combined all of the above and involved strength, aerobic, and anaerobic conditioning. There were also a number of small-sided rugby game sessions (Auckland only). As with FFIT, the exercise program was designed to ensure it was fun and varied; this was also a key recommendation that emerged from our focus groups. For the purpose of this pilot study no formal evaluation was undertaken to determine intervention fidelity.

#### Control condition

No lifestyle behavior information (e.g. advice about diet and/or exercise) or physical activity intervention was provided to control participants until the end of 12-week follow-up, when they were offered the 12-week RUFIT-NZ intervention. Men with undiagnosed high blood pressure (as identified at baseline measurements) were advised to consult with their general practitioner.

#### Potential for weight loss and change in lifestyle behaviors

Body weight in kg was measured as a preliminary primary outcome for a full trial. Bodyweight was measured using digital scales (Tanita UM-070 or Inbody 230, Biospace Co Ltd). Participants were dressed in light clothing and did not wear shoes. Height was measured with a stadiometer (Seca). Waist circumference was measured using a tape measure, while body fat was measured using bioelectrical impedance (ImpediMed DF50).

#### Health, fitness, and self-reported outcome measures

We assessed seated resting heart rate, and systolic and diastolic blood pressure, all measured using an automated sphygmomanometer (OMRON T9P Intellisense Blood Pressure Monitor or Omron Automatic Blood pressure monitor HEM-7322). Fitness was assessed by a 4 km cycle test. We also investigated adherence to recommended health guidelines measured as a binary variable using a self-reported composite health behavior score based on the European Prospective Investigation into Cancer (EPIC)-Norfolk Prospective Population Study [38]. Participants received a score from 0 to 4 (out of 4) based on the number of health guidelines they met. The health behaviors, scores, and outcome measures were smoking habit (1 = not currently smoking; 0 = had ≥1 cigarettes in past 7 days) assessed via a smoking history questionnaire [39], physically activity (1 ≥ 150 min/week of moderate to vigorous intensity physical activity [MVPA]; 0 ≤ 150 min/week of MVPA) assessed by the Godin Leisure Time Physical Activity Questionnaire [40], alcohol intake (1 indicates ≤13 units per week; 0 indicates ≥14 units per week) as measured by the Alcohol Use Disorders Identification Test Consumption (AUDIT C)] [41], fruit and vegetable intake (1 indicates ≥5 servings daily; 0 indicates ≤4 servings daily) from the NZ Health Survey [42]. Based on their score, participants were classified as ‘adherent’ if they scored 3 or more out of 4 and ‘non-adherent’ to current health guidelines if they scored 2 or less.

#### Feasibility outcomes and retention

Feasibility was assessed by recruitment and retention rates. Successful recruitment was defined as recruitment of at least 40 participants at each site over 1 month, while successful retention was defined as retention of at least 80% of participants who provided baseline measures. These parameters were set to inform recruitment targets and adherence for a larger trial. Intervention participants completed a feedback questionnaire at the 12-week follow-up assessment, which assessed (1) reasons for participating in the program, (2) reasons why participants did not attend all sessions, (3) whether the participants would recommend the program to other men, (4) what lifestyle behaviors men felt they had learned most about, and (5) what lifestyle behaviors they changed as a result of participating in the program.

#### Demographic information

Demographic information (data of birth to calculate age, self-reported ethnicity, employment status, highest level of education, marital status, and household income) was collected at baseline.

### Statistical analysis

As this was a feasibility study, no formal power calculation was conducted. The sample size was determined pragmatically to provide sufficient information on potential effects on key outcomes (weight and lifestyle variables), recruitment rates, sample variability, retention, and ability of the rugby clubs to host this number of participants. We aimed to recruit a maximum of 100 participants. Baseline demographic characteristics of all randomized participants were summarized descriptively, by intervention and control groups. Continuous variables were described as mean and standard deviation, or median and range. Categorical variables were described as frequencies and percentages.

For pre-specified primary and secondary outcomes (described above), descriptive summaries were presented at baseline and 12-week follow-up for the intervention and control group separately. The effect of the intervention on continuous outcomes at 12 weeks was evaluated using the analysis of covariance regression model adjusting for baseline outcome, age and ethnicity (Maori/Pacific versus Other). The adjusted treatment size was estimated with 95% confidence interval. Adjusted logistic regression was used to measure the treatment effect on the proportion of participants adherent to three or more healthy lifestyle behaviors at 12 weeks. As this was a pilot trial no imputation was undertaken for missing data. Due to the limited sample size, subgroup analyses were not conducted on each club separately. Statistical analyses were performed using SAS version 9.4 (SAS Institute Inc. Cary NC).

## Results

**Fig. 1:**
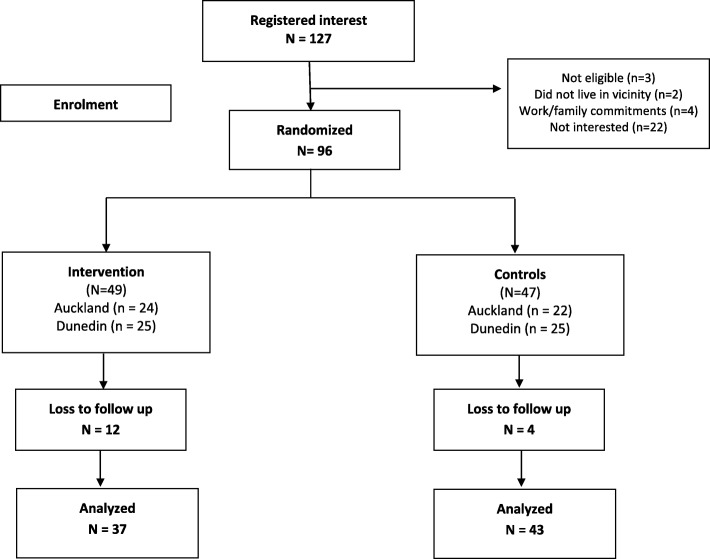
Study flow diagram

A total of 96 participants (*n* = 49 intervention; *n* = 47 controls) were recruited (*n* = 46 Auckland; *n* = 50 Dunedin), see Fig. 1 for the CONSORT 2010 flow diagram. Baseline data are presented in Table 3 for the 84 (87.5%) participants who completed the assessments (*n* = 45 intervention; *n* = 39 controls).

**Table 3:** Characteristics of Participants at Baseline

	Controls *N* = 47	Intervention *N* = 49
Participants		
Completed baseline assessment (n, %)	39 (83.0)	45 (91.8)
Demographics		
Age in years (mean, SD)	44.7 (8.9)	40.6 (8.9)
Ethnicity (n, %)		
NZ European	26 (66.7)	26 (57.8)
Māori	5 (12.8)	5 (11.1)
Pacific	5 (12.8)	8 (17.8)
Other	3 (7.7)	6 (13.3)
Marital status		
Living with partner	30 (68.1)	35 (67.3)
Separated/divorced	3 (6.8)	1 (1.9)
Never married	4 (9.0)	8 (15.3)
Refused to answer	2 (4.5)	7 (13.4)
Education (n, %)		
None	4 (10.2)	3 (6.6)
5th form qualification	5 (12.8)	2 (4.4)
6th form qualification	2 (5.1)	0 (0.0)
School qualification higher than 6th form	3 (7.6)	4 (8.8)
Other school qualification	3 (7.6)	1 (2.2)
National Certificate, Trade Certificate	4 (10.2)	8 (17.7)
Polytechnic/University below Bachelors degree	5 (12.8)	2 (4.4)
Bachelors degree	9 (23.1)	16 (35.5)
Degree higher than Bachelor	2 (5.1)	7 (15.5)
Other	1 (2.5)	1 (2.2)
Refuse to answer	1 (2.5)	1 (2.2)
Household Income (n, %)		
$70,000/year or less	13 (33.3)	23 (51.1)
More than $70,000/year	22 (56.4)	20 (44.4)
Don’t know/Refuse to answer	4 (10.3)	2 (4.4)

### Effect on weight and adherence to lifestyle behaviors

Weight, anthropometrics, physiological and lifestyle outcomes are reported in Table 4. A − 2.5 kg (95%CI -5.4 to 0.4) mean weight loss at 12 weeks favored the intervention group. There were statistically significant differences in waist circumference, resting heart rate, and diastolic blood pressure, improved cardiorespiratory fitness, and the proportion adherent (adjusted odds ratio 7.9; 95%CI 1.3 to 48.8) to lifestyle behaviors, which favored the intervention group. No statistically significant differences were observed for systolic BP, or percentage body fat.

**Table 4:** Anthropometric, health, fitness, and other self-reported outcomes at baseline and 12 weeks

Outcomes	Controls Baseline Mean (SD)	Intervention Baseline Mean (SD)	Controls 12 weeks Mean (SD)	Intervention 12 weeks Mean (SD)	Adjusted mean difference: intervention vs. control 95% CI	*P* value
Weight (kg)	110.9 (23.5)	114.7 (25.0)	111.6 (22.1)	113.3 (25.7)	−2.5 (−5.4 to 0.4)	0.09
Waist circumference (cm)	117.1 (14.5)	120.1 (16.9)	114.4 (14.7)	113.6 (17.2)	−3.5 (−5.1 to −1.9)	< 0.0001
% Body fat	34.1 (6.7)	33.9 (8.3)	36.5 (14.5)	32.3 (8.4)	−1.8 (−8.5 to 5.0)	0.598
Resting HR (bpm)	73.0 (10.2)	79.3 (16.5)	75.7 (12.2)	71.2 (12.3)	−6.7 (− 11.6 to − 1.8)	0.007
Systolic BP (mm Hg)	143.4 (14.9)	143.1 (16.9)	137.5 (14.8)	138.4 (18.0)	0.9 (−4.9 to 6.7)	0.769
Diastolic BP (mm Hg)	94.8 (11.2)	94.5 (13.9)	92.8 (9.8)	88.7 (15.7)	−4.2 (−8.2 to −0.2)	0.040
Fitness (4 km cycle test sec)	404.4 (52.0)	412.1 (62.0)	411.1 (53.7)	377.1 (91.1)	−26.2 (−53.2 to 0.8)	0.06
Adherent to 3 or more life-style behaviors						
Yes (n, %)	25/37 (67.6%)	22/42 (52.4%)	27/37 (73.0%)	33/35 (94.3%)	OR 7.9 (1.3 to 48.8)	0.03

Linear and Logistic regression models on key outcomes measured at 12 weeks. All models adjusted for age, Maori/Pacific ethnicity and baseline outcomes. Adjusted Odds Ratios (OR) reported for composite lifestyle score (Yes vs No). *SD* Standard Deviation; *CI* is Confidence Interval

### Feasibility issues

With respect to feasibility, 127 participants were assessed for eligibility, of whom 3 were excluded due to not meeting the inclusion criteria (either they were not overweight or they were already meeting the NZ physical activity guidelines). An additional 22 men registered but were not interested in the study once they received further information. Recruitment was completed within one month and the recruitment target of 90 participants (40 in Auckland and 50 in Dunedin) was exceeded (*n* = 96). The retention target of 80% was exceeded, with 97% of controls and 82% of intervention recipients who provided baseline data participating in final follow-up. Of the 37 intervention participants who completed final follow-up their main reasons for joining the RUFIT-NZ program included to get fitter (89%; 33/37), to improve their lifestyle (78%; 29/37), to lose weight (76%; 28/37), and for health reasons (49%; 18/37). Over a third of the men also reported joining the program because it was “with men like me” (35%; 13/37). The study procedures, including the randomization process and data collection methods, were deemed acceptable to the participants and the rugby clubs.

### Participant follow-up

Overall, 100% of the men from the intervention condition that were followed up reported they liked they program and would recommend it to other men. Further, 97% (36/37) said that the program helped them change their lifestyle behaviors, including being more physically active (92%; 34/37), eating more fruit and vegetables (78%; 29/37), eating less fatty foods (57%; 21/37), lowering stress levels (35%; 13/37), eating less salt (35%; 13/37), getting more sleep (30%; 11/37), and watching less TV (27%; 10/37). The main reasons for missing sessions included work commitments (41%; 15/37), family commitments (35%; 13/37), and health reasons (27%; 10/37); no men reported dislike of the RUFIT-NZ sessions or time constraints as reasons for not attending sessions.

## Discussion

The present study was designed to develop the RUFIT-NZ program, a healthy lifestyle intervention for men delivered via professional rugby clubs in NZ (the RUFIT-NZ program), and to determine its potential for influencing body weight and healthy lifestyle behaviors (physical activity, diet). Additional aims were to assess the feasibility and acceptability of RUFIT-NZ. Inspired by FFIT, RUFIT-NZ was developed by incorporating (1) feedback from potential participants and stakeholders, (2) advice from consultation with experts in the field, and (3) findings from a review of relevant NZ guidelines for weight management, physical activity and diet. The RUFIT-NZ program was successfully delivered over 12 weeks in two professional rugby clubs, with some evidence of positive effective effects observed for weight, waist circumference, resting HR and BP, cardiorespiratory fitness, as well as adherence to lifestyle guidance. The program was considered feasible to deliver, with good recruitment and acceptable retention rates. Consequently, results from this study confirm the feasibility and acceptability of the RUFIT-NZ program and indicate the need to proceed to a full RCT to determine its effectiveness.

### Strengths and limitations

This was the first randomized controlled pilot study to determine potential effect and the feasibility of a healthy lifestyle intervention designed specifically for the needs of men conducted through professional rugby in NZ. A number of validated outcome measures were assessed, and post-intervention participant evaluation of the RUFIT-NZ program provided information about acceptability. Further, the inclusion of two rugby clubs located in different parts of the country with substantially distinct ethnic compositions (Auckland has a larger proportion of Māori and Pacific peoples compared with Dunedin) [43], enhances the generalizability of the findings.

Findings from this study should be considered with the following limitations in mind. Approximately 17% (4/24) of participants randomized to the RUFIT-NZ intervention in Auckland did not attend the baseline measurement. Similarly 17% (8/47) of all participants randomized to the control condition did not attend the baseline assessment. It is not known why these participants withdrew before attending the baseline session; however, a definitive trial would address this by ensuring participants are randomized following baseline data collection.

This study took a pragmatic approach to its delivery of the intervention, thus there were some differences in how RUFIT-NZ was delivered between the Auckland and Dunedin sites. Due to logistical constraints specified by the rugby clubs and information provided during focus groups, a similar core educational content of the program was delivered across both sites; however the availability of sessions offered differed (e.g. the number and duration of sessions offered). For the present study, RUFIT-NZ coaches were given the materials for the education sessions, and tips on how to deliver it, but were given freedom to decide how to structure each of the physical activity sessions. In house training was provided to the RUFIT-NZ coaches; however this was not standardized across sites as the coaches were already trained to work with overweight men and knew how to effectively manage safety and injury prevention. A full trial would incorporate full and standardized training of the trainers and assess intervention fidelity. Finally, due to logistic constraints (time and financial limitations), a number of behavior change outcome measures relied on self-report, which may be subject to social desirability and recall biases.

### Comparison with previous research

The effect on body weight in this study was smaller than observed post-program (at 12 weeks) or at 12 months in the FFIT trial; however it is important to acknowledge that this study was not sufficiently powered to detect changes in body weight, nor was the timeframe (12 weeks) for assessment sufficiently long to determine effects on sustained weight loss. As a result of this pilot study we have made changes to the proposed full RCT to enhance fidelity of intervention delivery (training and monitoring of the trainers), and have included a greater emphasis on self-monitoring of weight and behavioural maintenance components to the intervention, which are likely to result in a larger effect. Positive effects on more proximal outcomes, including cardiorespiratory fitness, HR and BP, as well as adherence with lifestyle change were observed and highlight the potential positive health effects of this intervention.

### Feasibility, acceptability, and other outcomes

Due to the nature of the recruitment process, which included online Facebook advertising via promoted posts and advertising on the rugby club websites, we were unable to establish recruitment reach; however, we screened a total of 127 participants for eligibility, of whom 96 were deemed eligible and agreed to participate in the study. This met our criteria for recruitment feasibility and acceptability of the inclusion criteria. However there appeared to be a differential loss-to-follow up with a greater proportion of participants not completing follow-up assessments. As a result of this pilot study we have proposed greater contractual obligation with participants at the start of the future trial to ensure greater accountability to the RUFIT-NZ intervention. Feedback from participants was also positive highlighting key changes in behavior risk factors for obesity (changes to diet, alcohol consumption as well as physical activity).

### Content development

While inspired by FFIT program, formative work (focus groups and key informant interviews) highlighted some changes to ensure RUFIT-NZ resonated with the environment and culture of NZ. Changes included moving toward a more holistic perspective of health or ‘haoura’ in Māori. Hauora is a Māori philosophy of health unique to NZ. It comprises taha tinana (physical well-being), taha hinengaro (mental and emotional well-being), taha whanau (social well-being), and taha wairua (spiritual well-being). Where possible these perspectives were considered in the development of RUFIT-NZ. This approach was also reflected in the delivery of the Auckland-based sessions where partners of the men were encouraged to attend the education sessions, given that they are often the ones doing the food shopping for the family and preparing the meals; partners of some men did attend these sessions. Other nuances of RUFIT-NZ involved changing the eligibility criteria to include men with a lower BMI (≥25 kg/m^2^ versus ≥28 kg/m^2^), which is consistent with current definitions or threshold for overweight. The intervention also included classroom sessions on mindful eating, improving sleep, and reducing screen use and sedentary behaviors. Further, in contrast to FFIT, nutrition information was delivered by a nutritionist. This approach emerged from the focus groups as men stated they wanted nutrition information from a credible source who could provide answers to questions quickly.

### Future research

Despite the positive preliminary results from RUFIT-NZ, a definitive trial is warranted to determine the effects on weight and health outcomes at 12 months. Such a trial would include many of the original features of RUFIT-NZ but would include a much larger sample size, longer duration follow-up, standardized delivery of the intervention across sites with one session offered per week for 12 weeks, standardized training for coaches in the delivery of the intervention, monitoring of intervention fidelity, and the inclusion of a ‘light’ touch maintenance component to the program to ensure men continue the healthy lifestyle behavior change beyond the initial 12 week RUFIT-NZ intervention. Findings from this trial and others have relevance for men in other countries or regions with high rates of obesity including Tonga and other Pacific Islands, Mexico and South America, where sport is an important part of national identity.

## Conclusion

A pilot study of a healthy lifestyle intervention delivered via professional rugby clubs in NZ **(**RUFIT-NZ) demonstrated positive effects on weight, physiological outcomes, as well as adherence to lifestyle behaviors. Feasibility issues in terms of recruitment, retention, and participant acceptability were addressed and findings will be used to inform the design of a definitive trial.

## Abbreviations

AUDIT C: Alcohol Use Disorders Identification Test Consumption
BCT: Behavior Change Technique
BMI: Body Mass Index
CI: Confidence Interval
CONSORT: Consolidated Standards of Reporting Trials
EEPIC: European Prospective Investigation into Cancer
FAB: Food, Activity, Behavior
FFIT: Football Fans in Training
ITT: Intention To Treat
MVPA: Moderate to Vigorous Physical Activity
NZ: New Zealand
PAR-Q: Physical Activity Readiness Questionnaire
RCT: Randomized Controlled Trial
RUFIT-NZ: Rugby Fans in Training – New Zealand
SMART: Specific, Measurable, Achievable, Realistic, Timely
